# The Effect of Porcelain Firing and Type of Finish Line on the Marginal Fit of Zirconia Copings

**Published:** 2015-06

**Authors:** Mahroo Vojdani, Anahita Safari, Mina Mohaghegh, Soheil Pardis, Farideh Mahdavi

**Affiliations:** 1 Biomaterial Research Center, Dept. of Prosthodontics, School of Dentistry, Shiraz University of Medical Sciences, Shiraz, Iran;; 2 Dept. of Prosthodontics, School of Dentistry, Shiraz University of Medical Sciences, Shiraz, Iran;; 3 Dept. of Pathology, School of Dentistry, Shiraz University of Medical Sciences, Shiraz, Iran;; 4 Postgraduate Student of Prosthodontics, School of Dentistry, Shiraz University of Medical Sciences, Shiraz, Iran.

**Keywords:** Marginal fit, CAD/CAM, Zirconia, Full-ceramic crown, Porcelain veneering

## Abstract

**Statement of the Problem:**

Although all-ceramic restorations are broadly used, there is a lack of information concerning how their fit is affected by fabrication procedure and marginal configuration.

**Purpose:**

The purpose of this study was to evaluate the marginal fit of zirconia CAD/CAM ceramic crowns before and after porcelain firing. The influence of finish line configuration on the marginal fit was also evaluated.

**Materials and Method:**

Twenty standardized zirconia CAD/CAM copings were fabricated for chamfer and shoulder finish line designs (n=10). The marginal fit of specimens was measured on 18 points, marked on the master metal die by using a digital microscope. After the crowns were finalized by porcelain veneering, the measurements of marginal fit were performed again. The means and standard deviations were calculated and data were analyzed using student’s t-test and paired t-test (α=0.05).

**Results:**

There were significant differences between marginal fit of chamfer and shoulder finish line groups before and after porcelain firing (*p*= 0.014 and *p*= 0.000, respectively). The marginal gap of copings with shoulder finish line was significantly smaller than those with chamfer configuration (*p*= 0.000), but there were no significant differences between the two marginal designs, after porcelain firing (*p*= 0.341).

**Conclusion:**

Porcelain veneering was found to have a statistically significant influence on the marginal fit of zirconia CAD/CAM crowns. Both margin configurations showed marginal gaps that were within a reported clinically acceptable range of marginal discrepancy.

## Introduction

As far as esthetics and acceptable biocompatibility of dental restoration are concerned, all-ceramic crowns have recently gained large popularity. If made of high quality, all-ceramic restorations are difficult to be distinguished from unrestored adjacent teeth.[1]

Among the many ceramic systems that have been developed,[2-3] Yttria-stabilized polycrystalline tetragonal zirconia has become a popular form of dental restoration; mostly because of its notable characteristics including esthetics, excellent biocompatibility, low plaque accumulation, and high strength.[4] For the fabrication of zirconium oxide core, computer-aided design/ computer-aided manufacturing (CAD/CAM) is used by the system.[5] A compatible feldspathic translucent veneering porcelain (facing porcelain) is applied onto the white zirconia core to guarantee the excellent esthetics of the restorations.[4] This veneering process which includes a firing procedure (sintering) at high temperature (750-900^°^C) and subsequent cooling of the restoration, is carried out at least once, but usually 2-5 times.[6]

One of the most important standards in clinical assessment and success of fixed dental restorations is marginal fit of the crown.[7-10] In fact; marginal misfit has many severe outcomes which may induce prospective failure of the prosthesis.[9]Large marginal discrepancies make the luting agent to be disclosed within the oral environment. If the marginal gap is large, the cement will decompose rapidly as a result of oral fluids and chemomechanical forces.[11] This microleakage, in part, results in secondary caries, pulpal inflammation, and necrosis.[9-13] Inept marginal adaptation also causes plaque retention and compositional changes in the subgingival microflora, and consequently inflammation in gingival and periodontal tissues.[14] Finally, marginal misfit generates stress concentrations which may decrease the strength of the restoration.[15]

Marginal fit of the crown is defined as the gap between the prepared tooth and the intaglio surface of the restoration. Absolute marginal discrepancy is the linear distance between the cavosurface finish line of the preparation and the margin of the restoration.[16] This measurement displays the total misfit at the margin and is always considered as the largest measurement of the error at that point.[17] Mclean *et al.* defined clinically acceptable marginal discrepancies to be between 40 to 120 µm.[18] Previous studies have reported marginal discrepancy range of zirconia ceramic crowns to be 19 to 160 µm.[15, 19-23] However, there is limited studies on the marginal fit of zirconia-based materials in comparison with conventional ceramic or metal restorations.[24]

Given the importance of the fitting accuracy of restoration,[4] there has been much debate on the effect of veneering porcelain on all-ceramic restorations fit.[15, 19-20,25-27] To name a few, Balkaya *et al.* reported that the porcelain firing cycle has an influence on the marginal fit of In-ceram all-ceramic crowns.[15] Castellani *et al.* also pointed out that the marginal area of single crowns manufactured with different all-ceramic systems deforms significantly during the porcelain veneering process.[26] Contrary to these findings, Pera *et al.* found that the processes of firing and glazing of vitadur-N veneer did not alter the dimensional stability of In-ceram substructures.[19]

Moreover, the effect of the type of marginal design on the fitting precision of restoration should also be studied rigorously; for, there is no mutual agreement concerning ideal margin configuration of all-ceramic restorations. Researchers advocated either deep chamfer or rounded shoulder finish lines.[20, 28] Some studies on Procera ceramics[29] and zirconia ceramic crowns[30] suggested a significant difference in marginal gap between the two marginal designs. Based on their findings, rounded shoulder was identified to perform better. In contrast, some other studies illustrated that margin configuration had no significant difference on the marginal fit of ceramic crowns.[20, 31-32]

Marginal discrepancy can, in fact, be measured by using several methods such as direct view of the crown on a die, cross-sectional view, impression replica technique, and clinical examination.[33] The direct view, as used by the researchers of the current study, is a non-destructive technique which is frequently employed to measure the distortion during the manufacturing process of the restorations.[15]

The purpose of this study was to evaluate the marginal fit of zirconia CAD/CAM ceramic crowns before and after porcelain firing. The influence of finish line configuration on marginal fit was also evaluated. The null hypothesis was that no differences would be found in the marginal fit of zirconia CAD/CAM crowns before and after porcelain firing, and among different finish lines. 

## Materials and Method

*Fabrication of master dies*

**Figure 1:**
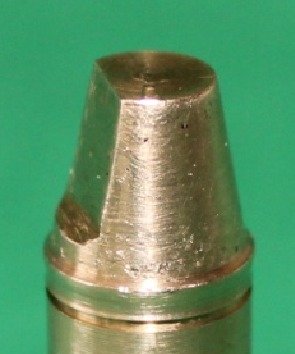
Brass master die

Brass master dies (Figure 1) were prepared for rounded shoulder and deep chamfer margins in a lathe (CNC350; Arix Co. Tainan Hesin, Taiwan). Preparation of master dies was done in accordance with the current standards of full-ceramic restorations.[1]

The preparation was standardized using a 1 mm wide smooth continuous margin, free of any irregularities, with occlusal convergence of 6 degrees with a height of 7 mm. A ledge was formed at the occluso-axial line angle to serve as anti-rotational feature. The measuring areas for evaluation of absolute marginal gap (AMG) were marked as 18 grooves at 20 degree intervals with a high speed handpiece (KaVo K9; KaVo dental GmbH, Biberach, Germany) and a diamond needle bur on a 2 mm groove below the margin. The sample size and the number of measurements per die were selected based on previous published studies.[15-16]

*Fabrication of the copings*

**Figure 2:**
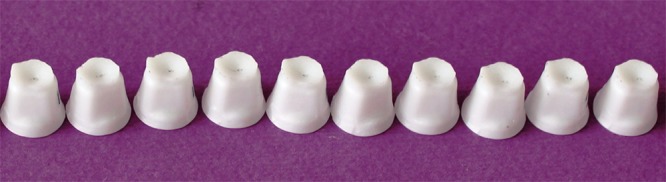
Fabricated zirconia CAD/CAM copings

The master dies were placed in a mold made of baseplate wax and checked with a surveyor (Ney Dental Surveyor; Dentsply, Ballaigues, Switzerland) to ensure its parallelism. The master dies were maintained in their place during acrylic packing by using wooden struts and sticky wax. Next, the mold was filled with auto-polymerized acrylic resin (DuraLay; Reliance Dental Co., Place Worth, IL). In this manner, the groove below the margin of the master die was 2 mm above the acrylic surface. Impressions of master dies were made out of additional putty and wash silicone materials (elite HD+; Badia Polesine, Zhermack Rovigo, Italy) in special trays (GC pattern resin; GC Corp., Tokyo, Japan). Twenty working dies (n=10 per each group) were fabricated using type *IV* dental stone (elite rock; Badia Polesine, Zhermack Rovigo, Italy). The stone dies were visually inspected for any possible irregularity by a single operator utilizing a binocular loupes (HEINE HR-C 2.5x; HEINE, Herrsching, Germany). The stone dies were coded and then scanned by a laser scanner (3Shape D810; 3Shape, Copenhagen K, Denmark) for digitizing the dies. The data were then transferred into a software (3Shape's CAD Design software; 3Shape, Copenhagen K, Denmark) in which the copings were designed with a thickness of 0.5 mm considering the 30 µm spacer 1 mm short of margin.[4] Copings were machined out of zirconium blanks (VITA In-ceram YZ-14; Vident, Germany) which were made of partially stabilized zirconium powder mixed with a binder in a milling machine (CORiTEC 340i; Imesicore GmbH, Eiterfeld, Germany) (Figure 2). Before sintering, the copings were steam cleaned. The machined copings, which had to be 25% larger than stone dies in order to compensate for the sintering shrinkage, were transformed back into their original size after the sintering. The copings were then seated on the master dies.

*Marginal discrepancy evaluation*

**Figure 3:**
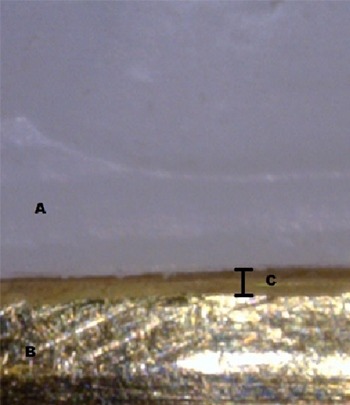
Captured image of coping-die interface (Zirconia coping (A), Brass master die (B), marginal gap (C))

The process of marginal discrepancy evaluation started with the copings being placed over the master dies using a special metallic device. To measure the marginal fit of the copings, the perpendicular measurement from the internal surface of the restoration margin to the most outer edge of the finish line of the preparation (AMG) was taken at 18 previously- marked points by use of a digital microscope (AM413FIT Dino-Lite Pro; Dino-Lite electronic Corp., Taipei, Taiwan). The microscope was mounted on a desktop stand (MS35B; Dino-Lite, Taipei, Taiwan), connected to a personal computer (PC) via USB 2.0 connection and photographed sequentially at 230 x magnification. High-resolution photographs were captured and displayed on the computer monitor. (Figure 3)

Then, the measurements were taken based on the produced images.

*Porcelain firing cycles*

**Figure 4:**
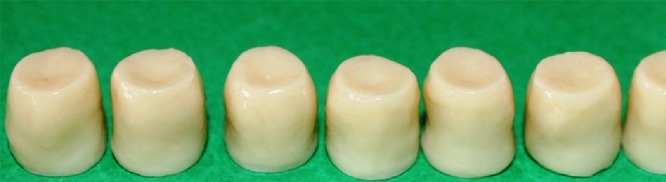
The completed crowns after porcelain firing

At this stage, the copings were prepared for porcelain application (Vita VM9; Vident, Germany). Porcelain application was done 0.5 mm short of margin.[24, 33] A silicone index was used to standardize the shape and the size of veneers. Next, the dentin and enamel porcelain were applied. (Figure 4) After each step, porcelain thickness was measured with a gauge (POCO 2N; Kroeplin, Schlüchtern, Germany). For all of the copings, porcelain application and firing cycles were done by a skilled technician based on the current standards. The marginal fit was measured again on the final master dies at the previously marked points.

*Statistical analysis*

**Figure 5:**
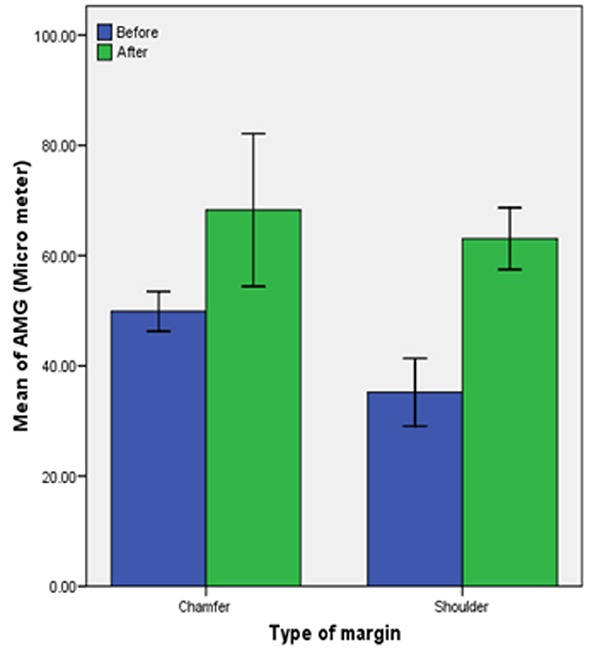
Mean values of marginal gap before and after porcelain firing

The means of different groups were compared using student’s t-test at the significant level of 0.05. Paired t-test was also performed to compare the amount of marginal fit before and after the veneering of porcelain within the same group. (Figure 5) All statistical analyses were performed using SPSS 16.0 for windows (SPSS 16.00 for windows; SPSS Inc, Chicago, USA).

## Results

Table 1 shows the means and standard deviations for the marginal gap of the specimens before and after porcelain firing in micrometers, sorted out by the margin configuration.

Different superscript letter in each row indicates significant difference by student’s test at α=0.05. 

There were significant differences between marginal fit of the two groups before and after porcelain firing (*p*< 0.05). The mean score of marginal gap in deep chamfer marginal design before and after the porcelain application was 49.8 µm and 68.2 µm, respectively. The mean score for marginal gap in rounded shoulder marginal design was 35.2 µm before and 63.06 µm after the porcelain application.

The marginal fit of shoulder copings was significantly better than chamfer copings (*p*= 0.000), but there were no significant difference between the two margins, after firing the porcelain (*p*= 0.341). These findings suggest that porcelain firing cycles change the marginal fit of shoulder copings more adversely. For both margin configurations, the marginal discrepancy of zirconia copings showed significantly smaller gaps than that of completed crowns. However, there was no significant difference between completed crowns of both chamfer and shoulder marginal designs.

**Table 1:** Absolute marginal gap of zirconia copings and crowns (µm)

	**Copings**		**Crowns**		**P value**
	**Mean**	**SD**	**Mean**	**SD**	
Chamfer	49.87 a	3.62	68.24 b	13.84	0.014
Shoulder	35.20 a	6.15	63.06 b	5.59	0.000

## Discussion

The results of this study strongly support rejection of the first part of the null hypothesis; since there was a significant difference in the marginal fit of zirconia CAD/CAM crowns before and after porcelain firing. However, no significant difference was observed between the two margin configurations of the finished crowns. Thus, data support acceptance of this part of the null hypothesis.

In the current investigation, the researchers used a single metal die for each margin configuration. The application of this single standard master die provided standardized preparation and direct comparison of marginal discrepancies and also precluded any wear from being formed during the manufacturing and measuring processes. In addition, it prevented luting of individual crowns onto the dies, an event which could affect the marginal fit because of probable variance in luting agent's viscosity and seating forces.[20]

Since porcelain contamination on the margin of copings might influence the accuracy of measurements, the application of porcelain was done 0.5 mm short of margin. In addition, this procedure finely restrained partial seating of the crowns which could occur as a result of intaglio surface contamination.[24, 34]

The obtained mean scores for marginal gap were 68 µm for chamfer and 63 µm for shoulder margin configurations; which were close to the results of similar previous studies. Hertlain *et al.*[35] explored the marginal fit of Lava CAD/CAM all-ceramic system with a chamfer preparation and reported the marginal adaptation to vary between 40-70 µm. Tao *et al.*[23] reported that the marginal gap of Cercon crowns ranged from 40 to 90 µm. Mirza Rustam Baig *et al.*[24] also examined marginal fit of Cercon zirconia crowns and suggested the overall mean marginal gap of 66.4 µm. Moreover, they reported that margin configuration did not significantly affect the marginal gap of complete coverage Cercon crowns. Still in another study, Bindl and Mormann[36] compared the marginal discrepancy using Cercon zirconia material with chamfer and shoulder finish lines and reported comparable results regarding both finish lines.

In a compelling study, Kyu-Bok Lee *et al.*[37] evaluated the marginal fit of conventional double layered CAD/CAM system (Porcera) on metal dies with 1 mm shoulder margin. They found that after the porcelain firing, the marginal discrepancy width of Porcera crowns (89.6±9.5 µm) demonstrated significantly larger gaps than that of Porcera copings (72.2±7.0 µm) (*p*< 0.05). This difference, which was also observed in our study, could be justified by the fact that the porcelain veneering procedure makes the particles of porcelains melt and gather to fill up the gaps. Therefore, the resulting contraction of porcelain mass imposes a compressive force on the coping.[38] The consequent deformation of coping spreads over the whole circumference of the margin under the pressure of contracting porcelain.[38] Nevertheless, it must be noted that the marginal openings of the crowns after the porcelain veneering are within clinically acceptable standards, and the amount of deformation does not interfere with the clinical application.

Considering the layered restorations, a positive thermal mismatch will always lead to formation of tensile stress within the framework. While the veneering ceramic is subjected to compressive forces, a negative mismatch will produce a completely reverse effect. We are well aware that dental manufacturers have come along all-ceramic systems which appear to have veneering ceramics of slightly lower coefficient of thermal expansion (CTEs) than that of the framework, resulting in a positive mismatch of the CTEs. This positive mismatch is expected to induce a beneficial compression stress on the veneering porcelain layer.[39] Aboushelib *et al.*[39]presumed that minimizing the thermal mismatch would be desirable, especially for all-ceramic zirconia restorations. However, Isgro *et al.*[40]declared that even a zero thermal mismatch value is not enough to predict compatibility between ceramic core and veneering porcelain. According to these reports, there are other factors that need to be considered, including viscoelastic behavior of the porcelain, repeated firings, and fast or slow cooling procedures.

It can be concluded from the outcome of the aforementioned studies that the distortion of marginal fit could be due to the shrinkage of porcelain as a result of coping distortion, CTE incompatibility of the core and the veneering porcelain, and porcelain contamination of the internal surface of the copings.

As mentioned previously, porcelain firing cycles change the marginal fit of shoulder copings more adversely compared with rounded chamfer copings. The authors believe that this phenomenon occurs because the chamfer finish line has some length on axial wall of the preparation, so the closing of margin i more probable along this length. On the other hand, shoulder margin has a butt joint form, without any length on axial wall. This is why if any distortion happens due to porcelain firing, it will affect the whole marginal gap. In agreement with the current study, Pera *et al.*[19] that evaluated the marginal adaptation of porcelain ceramic crowns reported improved marginal fit of In-Ceram crowns fabricated on chamfer compared with shoulder finish line, although they did not explain the cause.

Certainly, this study was not free of limitations. Some of these restrictions are discussed as follows. First, marginal fit was measured in this experimental design; however, the internal fit of the crowns was not. The reason was that measuring the internal fit of the crowns required the crowns to be cemented and the specimens to be sectioned. Second, all copings were produced and tested under ideal conditions, which may not reflect the conditions which can be seen in daily clinical practices. Third, the copings were not subjected to mechanical and thermal cycling; while thermo mechanical cycling is one of the most important factors which affects the long-term success of the restoration.[41-42] Finally, although brass dies were used for measurement, use of human natural teeth would be more ideal.

## Conclusion

Within the limitations of this study, the following conclusions could be drawn:

1. Porcelain veneering showed to have a statistically significant influence on the marginal fit of zirconia CAD/CAM crowns.
2. There were no significant differences between completed crowns of chamfer and shoulder margins.
3. Both margin configurations demonstrated marginal gaps that were within a reported clinically acceptable range of marginal discrepancy.
